# Cytoplasmic ADP-ribosylation levels correlate with markers of patient outcome in distinct human cancers

**DOI:** 10.1038/s41379-021-00788-9

**Published:** 2021-03-19

**Authors:** Fabio Aimi, Holger Moch, Peter Schraml, Michael O. Hottiger

**Affiliations:** 1 University of Zurich (UZH), Department of Molecular Mechanisms of Disease (DMMD), Zurich, Switzerland; 2 University of Zurich and University Hospital Zurich (USZ), Department of Pathology and Molecular Pathology, Zürich, Switzerland

**Keywords:** Prognostic markers, Tumour biomarkers, PolyADP-ribosylation

## Abstract

ADP-ribosylation (ADPR) is a posttranslational modification whose importance in oncology keeps increasing due to frequent use of PARP inhibitors (PARPi) to treat different tumor types. Due to the lack of suitable tools to analyze cellular ADPR levels, ADPR’s significance for cancer progression and patient outcome is unclear. In this study, we assessed ADPR levels by immunohistochemistry using a newly developed anti-ADP-ribose (ADPr) antibody, which is able to detect both mono- and poly-ADPR. Tissue microarrays containing brain (*n* = 103), breast (*n* = 1108), colon (*n* = 236), lung (*n* = 138), ovarian (*n* = 142), and prostate (*n* = 328) cancers were used to correlate ADPR staining intensities to clinico-pathological data, including patient overall survival (OS), tumor grade, tumor stage (pT), lymph node status (pN), and the presence of distant metastasis (pM). While nuclear ADPR was detected only in a minority of the samples, cytoplasmic ADPR (cyADPR) staining was observed in most tumor types. Strong cyADPR intensities were significantly associated with better overall survival in invasive ductal breast cancer (*p* < 0.0001), invasive lobular breast cancer (*p* < 0.005), and high grade serous ovarian cancer patients (*p* < 0.01). Furthermore, stronger cytoplasmic ADPR levels significantly correlated with early tumor stage in colorectal and in invasive ductal breast adenocarcinoma (*p* < 0.0001 and *p* < 0.01, respectively) and with the absence of regional lymph node metastasis in colorectal adenocarcinoma (*p* < 0.05). No correlation to cyADPR was found for prostate and lung cancer or brain tumors. In conclusion, our new anti-ADP-ribose antibody revealed heterogeneous ADPR staining patterns with predominant cytoplasmic ADPR staining in most tumor types. Different cyADPR staining patterns could help to better understand variable response rates to PARP inhibitors in the future.

## Introduction

Some of the most recently approved drugs for the treatment of breast [1, 2] and ovarian [3, 4] cancer are poly-(ADP-ribose) polymerases (PARP) inhibitors (PARPi). Two breakthrough studies in preclinical models demonstrated the effectiveness of PARPi in the treatment of these cancers by showing synthetic lethality between the PARPi and the mutations in *BRCA1*/2 encoding components of homologous recombination-mediated DNA repair (HRR) pathways [5, 6]. Recently, the beneficial effect of Olaparib (PARPi) in treating metastatic castration-resistant prostate cancer patients was reported in a profound clinical study [7]. The use of PARPi is also considered in clinical trials for the treatment of several other tumor types, including lung cancer [8], pancreatic cancer [9], melanoma [10], glioblastoma [11], colorectal cancer [12–14], and renal cell carcinoma [15, 16], either alone or in combination with other drugs, and sometimes even independently of the *BRCA* status [17]. However, besides these encouraging results, the use of PARPi remains clinically relegated to few cancer types and their administration relays mainly on patient stratification based on “BRCAness” [18], obtained by gene mutation analysis of *BRCA1/2* and its modulators, without considering the actual PARP activities. Mechanistically, currently approved PARP inhibitors inhibit the activity of some ADP-ribosyl transferases (ARTs), also known as PARPs [19]. ARTs and their counterparts, ADPr-glycohydrolases, modulate the levels of ADP-ribosylation (ADPR), a reversible posttranslational modification (PTM) [20], by transferring ADP-ribose (ADPr) moieties from Nicotinamide Adenine Dinucleotide (NAD^+^) to specific amino acid acceptor sites on target proteins [21]. ADPR exists in two forms, namely: mono-ADP-ribosylation (MARylation), which consists of only one ADPr monomer linked to the target protein, and poly-ADP-ribosylation (PARylation), a chain(s) of ADPr moieties extending from the MARylated form of the target protein [20]. PARylation was intensively studied in the last decades and was shown to be involved in transcription control, protein degradation, chromatin organization, and DNA repair [6, 22]. MARylation has been reported in cell-based assays involved in tumorigenesis since ARTs known to only MARylate proteins also regulate several cellular processes critical for cancer cells, such as cell proliferation, apoptosis, growth, and metabolism [23]. Moreover, some of these ARTs were also reported to modulate the activity of proteins involved in molecular pathways that are frequently dysregulated in cancer cells, such as c-Myc and NF-κB [24, 25].

So far, MARylation has not been investigated by immunohistochemistry on patient histological sections due to the missing tools. The main reason for this lack can be ascribed to the difficulties encountered in the chemical synthesis of ADP-ribosylated peptides required for the host immunization and the generation of antibodies. Indeed, the chemical nature of the linkage and the variety of modified acceptor amino acids requires long, complicated synthesis processes that yield only low amounts of peptides [26, 27]. Therefore, so far, only few studies have investigated endogenous ADPR (MAR and PAR) as a potential biomarker in oncology [23, 28, 29]. One of these [29] recently provided evidence by immunoblotting that PARP1/ARTD1-dependent ADPR levels correlate with clinical outcome in high grade serous ovarian cancer patients, whereas the other studies mainly relayed on mass spectrometry-based approaches and their results about the role of ADPR in cancer are partially contradicting [23, 28]. However, a comprehensive cancer study about ADPR (MAR and PAR) that particularly focuses on the analysis of cellular staining patterns in different human tumors is still missing.

Following an established methodology [30], we recently successfully synthesized ADP-ribosylated peptides and produced a polyclonal antibody that detects both MARylated and PARylated proteins in Westernblot and Immunfluorescence independently of the binding site and without cross-reacting with other posttranslational modifications [31, 32]. By immunohistochemical staining of various tissue microarrays (TMAs) using this new anti-ADPr antibody, we investigated the cellular staining patterns of ADPR in different cancer types, as well as their relevance for tumor progression.

## Material and methods

### Cell culture and pellets

HeLa cells (Kyoto, ATCC, CCL-2) were cultured in Dulbecco’s modified Eagle’s medium (DMEM), supplemented with 10% fetal calf serum (FCS) and 1% penicillin/streptavidin at 37 °C with 5% CO_2_ until 80% confluency. Nuclear ADP-ribosylation was induced in HeLa cells by treatment with 1 mM H_2_O_2_ in PBS containing 1 mM MgCl_2_ for 10 min. Cell pellets were obtained after coincubation with human plasma in presence of thrombin and consequently fixed with 4% formalin, dehydrated, and paraffinized.

### Patient tissue samples and tissue microarray construction

Formalin fixed and paraffin embedded tumor samples were retrieved from the archives of the Department of Pathology and Molecular Pathology, University Hospital Zurich (Zurich, Switzerland) between the years 1993 and 2013, thus the patients were not treated with approved PARP inhibitors. For each tumor, one representative tumor tissue block was re-evaluated using hematoxylin and eosin-stained sections. Only those cases with representative tumor regions that contained at least 70% tumor cells were selected for tissue microarray (TMA) construction. All tumors were reviewed by pathologists of the Department of Pathology and Molecular Pathology specialized in their field. Classification, grading, and staging were performed according to current TNM and WHO classification. The TMAs contained 1108 breast carcinomas (three TMAs 336 + 344 + 428) [33], 142 ovarian carcinomas [34], 328 prostate carcinomas [35], 138 lung carcinomas [36], 236 colorectal carcinomas (two TMAs 101 + 135) [37], and 103 brain tumors [38]. The TMAs were constructed as described [39] and each punch had a diameter of 0.6 mm. This study was approved by the local commission of ethics (BASEC-Nr_2016-00811).

### Immunohistochemistry (IHC) staining

In total, 2.5 μm sections of TMAs and paraffinized cell pellets were placed on glass slides and immunohistochemically stained using Ultra Discovery (Ventana, Roche Diagnostics, Rotkreuz, Switzerland). TMA sections were pretreated with Tris-EDTA-Borate Buffer of pH 9 at 95 °C for 60 min (CC1 standard protocol, Ventana) and incubated with anti-ADPr antibody [31, 32] (rabbit, diluted 1:500 in Bond medium) for 44 min at 36 °C (Discovery Ultra, Ventana). ADPr was made visible using the UltraMap-Rabbit DAB detection kit (Ventana). Nuclear counterstaining was performed with hematoxylin. The polyclonal anti-ADPr antibody is not commercialized and it is available upon request if used only for academic purposes.

### Scoring of the IHC staining

For staining evaluation, stained slides were scanned using a NanoZoomer (Hamamatsu Photonics, Shizuoka, Japan). The images were analyzed with ObjectiveView^TM^ software. Tumors were scored as strong (+3), moderate (+2), or weak/negative (+1) for cytoplasmic ADPR (cyADPR) in the tumor cells. ADPR in the epithelium of normal tissue samples and nuclear ADPR signal in cancer biopsies among the TMAs were considered as positive reference for ADPR staining. The scores were blindly double-checked by an independent pathologist.

### Statistical analysis

The software Graphpad Prism 8 was used to run statistical analysis. The patient survival was reported with Kaplan–Meier chart and assessed with Mantel–Cox statistical analysis. Correlation of ADPR scores with pathological parameters was assessed by contingency analysis, chi-square test with a confidence interval (CI) of 95%. Univariate and multivariate analysis were performed with R Studio software. Cox proportional model was used for the statistical analysis as described [40]. Differences were considered significant when *p* value < 0.05.

### Immunofluorescent (IF) staining

In total, 2.5 μm sections of TMAs and paraffinized cell pellets were placed on glass slides and dried for 10 min at 60 °C, dewaxed for 10 min in HistoChoice Clearing Agent (Sigma, H2779), and rehydrated by consecutive washes in ethanol: 100%, 95%, 70%, and 40% v/v for 30 sec each. Heat-induced antigen retrieval was performed at 95 °C for 40 min with Dako Target Retrieval Solution of pH 9 (Dako, S2367). After cooling at room temperature, the slides were incubated 2.5% BSA (Fluka Analytical, 05488) in TBST 0.1% for 1 hour at room temperature. Anti-ADPr [31, 32] (rabbit, 1:500 dilution) and anti-ATP5a antibody (Abcam, mouse, 1:250) diluted in the blocking solution were incubated overnight at 4 °C. Secondary antibody Alexa Fluor 488 AffiniPure goat anti-mouse (Jackson ImmunoResearch, 115–545–003, 1:500 dilution) and Cy3 AffiniPure goat anti-rabbit IgG (Jackson ImmunoResearch, 111–165–144, 1:250 dilution) were incubated for 2 hours at room temperature. Nuclear counterstaining was performed DAPI (Biolegend, 422801, 1:10,000 dilution). Microscope cover glasses (Menzel–Glaser, 7001023) were mounted with Mowiol mounting medium.

### Fluorescent and confocal microscopy

Fluorescent microscopy images were acquired with Leica CTR6000, 63x magnification, 250 and 400 ms exposure time (for DAPI and ADPR respectively), 100% FIM. Slides stained without the primary antibody were used as technical negative control. Focal plan was established on DAPI signal.

Confocal microscopy images were acquired with the automated CLSM––Leica SP8 upright confocal laser scanning microscope equipped with four solid state diode lasers (405, 488, 522, and 638 nm), using an HCX PL APO CS2 63x immersion oil objective present at the University of Zurich Center for Microscope and Image Analysis (ZMB). Nuclear focus was used to establish the focal plane.

The images were analyzed with the Leica software.

## Results

### Immunostaining with the anti-ADPr antibody is repeatable and detects ADP-ribosylation in different cellular compartments

To investigate the possible implication of ADP-ribosylation (both MAR- and PARylation) in cancer we performed a comprehensive study by immunohistochemically staining TMAs with a recently developed anti-ADP-ribose (ADPr) antibody that recognizes both MAR- and PARylation [31, 32]. To first characterize the ADPR staining patterns of the antibody for immunofluorescence (IF) and immunocytochemistry (ICC) we stained HeLa cell pellets either untreated or incubated with hydrogen peroxide. Both methodologies revealed that untreated cells were characterized by a diffuse cytoplasmic ADPR signal, whereas the H_2_O_2_ treatment, known to induce nuclear ADP-ribosylation [32], led to the detection of strong nuclear ADPR signal (Suppl. Figs 1, 2). Moreover, the IF and ICC stainings of the cell pellets with the anti-ADPr antibody performed in different days were repeatable and showed comparable signal intensities and distributions (Suppl. Figs 1, 2). Next, we optimized the immunohistochemical staining on several tumors included in a test tissue microarray (TMA). Also here, the ADPR stainings were consistent in terms of signal intensity and distribution (i.e., pattern) in different repetitions (Suppl. Fig. 3).

### Differential ADP-ribosylation is predominantly observed in the cell cytoplasm

**Fig. 1:**
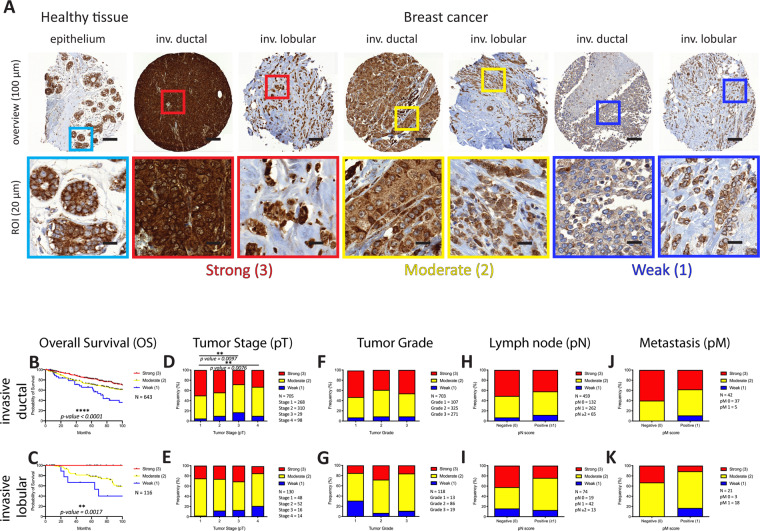
Analysis of the ADPR signal intensities in breast cancer. **A** IHC staining of a breast cancer TMA using the anti-ADPr antibody. cyADPR staining intensity scores: strong (red, 3), moderate (yellow, 2), and weak/negative (blue, 1). On the top, selected TMA cores (scale bar 100 µm, 10x), on the bottom, ROIs (scale bar 20 µm, 40x). Kaplan–Meier survival plot of (**B**) invasive ductal breast cancer (Mantel–Cox test, *p* value < 0.0001, *N* = 643) and (**C**) invasive lobular breast cancer (Mantel–Cox test, *p* value = 0.0017, *N* = 116) with patients stratified based on the cyADPR signal intensity scores. Contingency analysis to assess the association between cyADPR scores and the tumor stage (pT 1–4), the tumor grade (1–3), regional lymph node metastasis (pN 0–2), and the presence of distant metastasis (pM 0–1) in invasive ductal breast cancer (**D, F, H, J**) and in invasive lobular breast cancer (**E, G, I, K**).

We started our comprehensive tumor ADPR study by immunohistochemically staining three breast cancer TMAs using the anti-ADPr antibody according to our optimized staining protocol. Overall analysis of the signal localization and intensity revealed that a very weak homogenous nuclear ADPR signal (nuADPR) staining was observed only in rare cases of healthy tissues and for only a few breast cancer biopsies (12 out of 150) (Supplementary Fig. 4). Furthermore, intra-nuclear foci were observed in many cancer cells and in nearly all initially analyzed biopsies, but the weak ADPR signal staining did not allow to discriminate and score the signal intensities (data not shown). On the other hand, cytoplasmic ADPR (cyADPR) signal was observed in all the biopsies with different intensities (Fig. 1A). Thus, we decided to focus our study exclusively on the cytoplasmic ADPR (cyADPR) signal intensities.

### Strong cytoplasmic ADPR correlates with better outcome in breast cancer patients

Cytoplasmic ADPR signal intensities in breast cancer biopsies were scored using three categories (3: strong, 2: moderate, and 1: weak/negative) and subsequently correlated to patient clinical data including overall survival (OS), nuclear differentiation grade, tumor stage (pT), regional lymph node metastasis (pN), and distant metastasis (pM). In invasive ductal breast cancer (IDC), strong cyADPR intensity significantly correlated with better patient overall survival, whereas a weak/negative cyADPR signal intensity was associated with a poor patient prognosis (*n* = 643, *p* < 0.0001, Fig. 1B). Also, in invasive lobular breast cancer (ILC) biopsies, similarly to the results mentioned before, strong cyADPR was significantly associated with better patient outcome (*n* = 116, *p* = 0.0017, Fig. 1C).

In IDC, we observed that pT1 tumors displayed in 50% of the cases a strong cyADPR intensity, whereas in pT3 and pT4 cases strong cyADPR signal intensity significantly dropped to 28% and 33%, respectively (*n* = 705, *p* = 0.0097 and 0.0076, Fig. 1D). In ILC, we observed a gradual increase of weak/negative cyADPR intensity scores incidence from pT1 (2%) to pT4 (21%) but this difference was not statistically significant (*n* = 130, Fig. 1E). No significant correlation was obtained between cyADPR intensities and nuclear differentiation grade in both IDC (*n* = 703, Fig. 1F) and ILC (*n* = 118, Fig. 1G). In contrast, an interesting tendency although not statistically significant between decreased cyADPR levels and the presence of metastasis in regional lymph node (pN1/2) was noticed for IDC (*n* = 459, Fig. 1H) but not for ILC (*n* = 74, Fig. 1I). No correlation was obtained for cyADPR intensities and the presence of distant metastasis (pM) neither in IDC (*n* = 42, Fig. 1J) nor in ILC (*n* = 22, Fig. 1K).

Multivariate analysis assessed with the Cox multivariate regression test (test likelihood ratio *p* = 8e–10) revealed that in IDC (*n* = 411, number of events = 127) cyADPR levels (*β* = −0.25, CI = 95%, HR = 0.78, *p* = 0.059) were not independent from tumor stage (*β* = 0.43, CI = 95%, HR = 1.53, *p* < 0.0001), nodular stage (*β* = 0.26, CI = 95%, HR = 1.30, *p* = 0.0348), and tumor grade (*β* = 0.49, CI = 95%, HR = 1.63, *p* = 0.0018) (Table 1). In ILC, due to the lower number of cases (*n* = 51) and events (*n* = 15) the multivariate analysis assessed with the Cox multivariate regression test (test likelihood ratio *p* = 0.002), did not yield significant outcome as obtained in IDC (Table 1). The data, however, indicate a trend, which is similar to that seen in IDC.

**Table 1:** Multivariate analysis of cyADPR and clinicopathological factors in breast cancer.

MULTIVARIATE ANALYSIS						
	Invasive ductal breast cancer (IDC) (*N* = 411, number of events = 127)			Invasive lobular breast cancer (ILC) (*N* = 51, number of events = 15)		
	Beta coeff.	HR (95% CI for HR) (lower 0.95–upper 0.95)	Wald test *p* value	Beta coeff.	HR (95% CI for HR) (lower 0.95–upper 0.95)	Wald test *p* value
cyADPR	−0.25	0.78 (0.60–1.01)	0.0589 (.)	−0.87	0.42 (0.12–1.42)	0.1633 ()
pT	0.43	1.53 (1.29–1.83)	<0.0001 (***)	0.54	1.71 (0.94–3.12)	0.0772 (.)
pN	0.26	1.30 (1.02–1.65)	0.0348 (*)	0.84	2.33 (0.97–5.55)	0.0571 (.)
Grade	0.49	1.63 (1.20–2.22)	0.0018 (**)	0.83	2.29 (1.01–5.19)	0.0472 (*)

Breast cancer patients divided in the two subgroups: invasive ductal (IDC) and lobular (ILC). Clinicopathological factors: cytoplasmic ADPR (cyADPR), tumor stage (pT), lymph node metastasis (pN) and tumor grade. *Beta coeff.* Beta coefficient, *HR* hazard ratio, *CI* confidence interval. Significance for each single factor assessed with Wald test. Significance codes: *p* < 0.001 (***), 0.001 < *p* < 0.01 (**), 0.01 < *p* < 0.05 (*), 0.05 < *p* < 0.1 (.), *p* > 0.1 (). Significance for the multivariate analysis assessed with Likelihood ratio test, Wald test and Score logrank test. Significance: *p* < 0.05.

### Strong cytoplasmic ADPR correlates with a better prognosis in high grade serous ovarian cancer

**Fig. 2:**
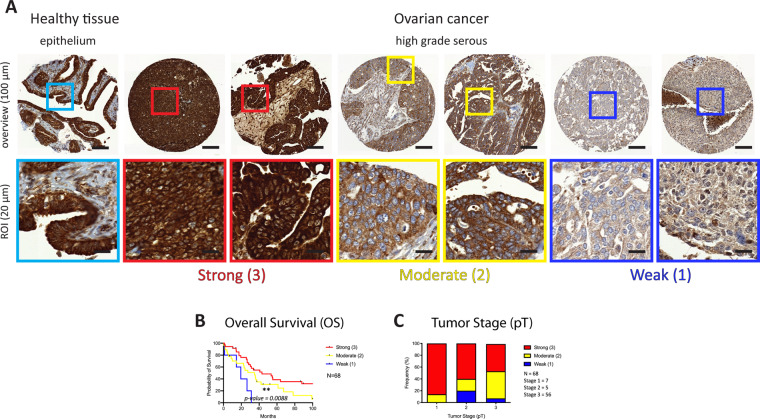
Analysis of the ADPR signal intensities in ovarian cancer. **A** IHC staining of an ovarian cancer TMA using the anti-ADPr antibody. Staining intensity scores: strong (red, 3), moderate (yellow, 2), and weak/negative (blue, 1). On the top, selected TMA cores (scale bar 100 µm, 10x), on the bottom, ROIs (scale bar 20 µm, 40x). **B** Kaplan–Meier survival plot of high grade serous ovarian cancer (Mantel–Cox test, *p* value = 0.0088, *N* = 68) with patients stratified based on the cyADPR signal intensity scores. **C** Contingency analysis to assess the association between cyADPR scores and the tumor stage (pT 1–4) in high grade serous ovarian cancer.

To assess whether cyADPR levels have prognostic impact in ovarian cancer, we analyzed 142 ovarian tumors for cyADPR signal intensities (Fig. 2A). By distinguishing between the ovarian cancer subtypes high grade serous (*n* = 68), clear cell (*n* = 26), endometrioid (*n* = 29), and mucinous (*n* = 14), we observed a significant correlation between high cyADPR levels and prolonged patient survival for high grade serous OC, the most abundant ovarian cancer subtype in the TMA (*p* = 0.0088, Fig. 2B). We also observed reduced cyADPR intensity in advanced tumor stage (pT) however, this tendency was not statistically significant (*n* = 68, Fig. 2C).

### Cytoplasmic ADPR signal intensities do not associate with prostate, lung, and brain cancer patient prognosis

Recent studies indicate the importance of ADPR in prostate [41, 42], lung [43, 44], and brain cancer [45]. We therefore analyzed cyADPR intensities of these cancer types using the weak, moderate, and strong cyADPR intensity scoring. Although staining differences could be observed, in all three tested cancer types, there were no significant correlations between cyADPR signal intensities and overall survival (OS), tumor stage (pT), differentiation grade or the metastasis presence (pN and pM), when data were available (Supplementary Figs 5, 6 and 7). Interestingly, lung adenocarcinomas with weak cyADPR signal intensities showed a trend to shorter patient survival, whereas this trend was not observed in lung squamous cell cancer.

### Weak cytoplasmic ADPR signal intensity correlates with late tumor stages and with lymph node metastasis in colon adenocarcinoma

**Fig. 3:**
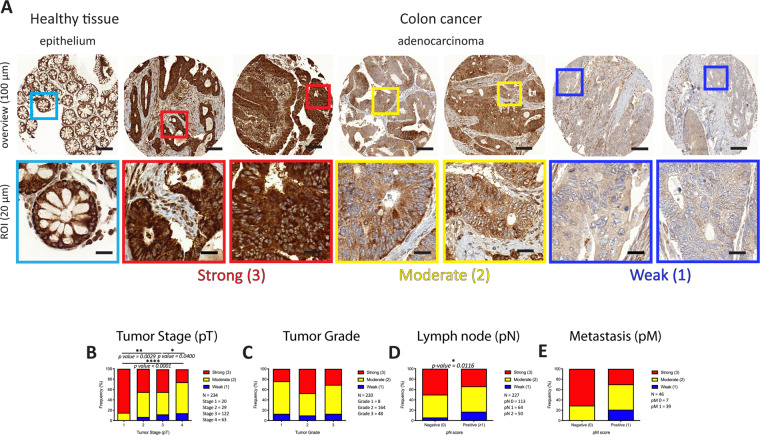
Analysis of the ADPR signal intensities in colon cancer. **A** IHC staining of a colon cancer TMA using the anti-ADPr antibody. Staining intensity scores: strong (red, 3), moderate (yellow, 2), and weak/negative (blue, 1). On the top, selected TMA cores (scale bar 100 µm, 10x), on the bottom, ROIs (scale bar 20 µm, 40x). Contingency analyses to assess the association between cyADPR scores and (**B**) the tumor stage (pT 1–4), (**C**) the tumor grade (1–3), (**D**) regional lymph node metastasis (pN 0–2), and (**E**) the presence of distant metastasis (pM 0–1).

Recently an association between ADPR and colon cancer development was suggested [46, 47]. We investigated cyADPR prognostic relevance in colon adenocarcinoma by analyzing cyADPR signal intensities in 256 biopsies allocated to two TMAs (Fig. 3A). Since patient survival data were lacking for the majority of the cases for this tumor type, it was only possible to associate the cyADPR scores to the classical prognostic markers tumor stage (pT), grade and metastasis (pN and pM). Interestingly, colon adenocarcinomas in pT1 were characterized in 85% by a strong cyADPR staining, whereas pT3 and pT4 tumors were characterized in 43% and 25%, respectively, by a strong cyADPR staining intensity. Together these data indicated a statistically significant decrease of cyADPR staining during tumor progression (Fig. 3B). No significant correlation was obtained between cyADPR intensities and nuclear differentiation grade (*n* = 220, Fig. 3C). Next, cyADPR signal intensity was correlated with the presence of metastasis. Half of the tumors lacking regional lymph node metastasis (pN0) had strong and only 6% weak/negative cyADPR signal intensity, whereas 34% of tumors with regional lymph node metastasis (pN ≥ 1) presented with a strong and 17% with a weak/negative cyADPR staining (*p* = 0.0116, *n* = 227, Fig. 3D). No significant correlation was obtained between cyADPR intensities and the presence of distant metastasis (pM) (*n* = 46, Fig. 3E).

### Mitochondrial ADP-ribosylome is the major source of cytoplasmic ADPR in selected tumor types

To better identify the cellular location of the cyADPR, we colocalized by confocal immunofluorescent microscopy the ADPR signal with an organelle-specific marker in different patient biopsies. Since recent studies showed mitochondria ADPR as major source of cellular ADP-ribosylome [48, 49], we co-stained breast, colon, lung, and ovarian cancer biopsies using the anti-ADPr antibody and an antibody specific for the mitochondrial marker ATP5a. We observed that the ADPR signal was frequently but not exclusively overlapping with the mitochondrial marker ATP5a, indicating that mitochondrial ADP-ribosylated proteins is one of the major sources of cytoplasmic ADP-ribosylome in these tumor types (Supplementary Fig. 8).

## Discussion

In this study, we describe heterogeneous ADPR staining patterns in ovarian, lung, breast, prostate, and colorectal adenocarcinomas and different brain tumors by using a newly developed anti-ADP-ribose (ADPr) antibody. Loss of cyADPR signal was associated with advanced tumor stage, lymph node metastasis or shorter overall survival only in specific adenocarcinoma types.

PARP inhibitors are currently used in breast and ovarian cancer therapies [2, 50, 51] and considered in clinical studies for the treatment of other frequently diagnosed tumor types (e.g., brain, colon, lung, and prostate [7, 41–47]). PARPi target and inhibit some ARTs, among which PARP1/ARTD1 is the most abundant. Since PARP1/ARTD1 is mainly localized in the nucleus [6, 22], so far, the attention of the ADP-ribosylation studies was mostly on nuclear ADPR. In this study, we identified the presence of a homogeneous and strong nuclear staining in however a small number of breast cancer biopsies. A deeper analysis revealed in many nuclei the presence of foci in the presence of otherwise weak overall ADPR staining. The number of foci most likely reflects the activity of ARTs involved in nuclear ADPR, and therefore their quantification might be highly informative. Thus, an extended analysis with confocal microscopy and supported by specific softwares might reveal the prognostic relevance of ADPR nuclear foci in different cancer types in the future.

Despite of the importance of nuclear ADPR, we exclusively focused here on cyADPR. We provide evidence that cyADPR is reduced in advanced breast, ovarian, and colorectal cancer with higher stages and lymph node metastases. Therefore, strong cyADPR signal intensity correlated with a better prognosis in breast and high grade serous ovarian cancer (with a similar trend in lung adenocarcinoma). Although the prognostic relevance of cyADPR staining was lost in multivariate analysis, our data indicated that in some tumor types cyADPR levels decrease during tumor progression. The reason for this phenomenon might be connected to different factors. First, the alteration during tumor development of cell metabolism and NAD^+^ availability as ADPR reaction substrate, which was shown to exert a protective effect against oncogenesis [52, 53], and alternatively, the modulation during tumorigenesis of the presence/activity of enzymes responsible for ADPR. Expanded investigations about the NAD^+^ homeostasis and the enzyme expression levels will provide further insights for the importance of ADPR levels during cancer development.

A very recent study underlined a correlation between ADPR levels and clinical outcome in a small cohort (*n* = 34) of ovarian cancer patients [29]. Differently than what we observed, that study indicated a negative correlation between the PARP1/ARTD1-dependent ADPR levels and the clinical outcome of high grade serous ovarian cancer patients [29]. This difference might be linked to different factors besides the cohort size. First, the tools (antibody or binding reagent) used to assess ADPR levels were different and might prefer ADPr moieties linked to specific amino acid acceptor sites thus reporting only a partial overview of the cellular ADP-ribosylome. Second, the ADPR-detecting tools used in these two studies were employed with different methodologies: immunoblot or immunohistology. Unfortunately, since the ADPR binding reagents used in the immunoblot-based study were not validated for IHC, a direct comparison on patient histological sections was not possible. Third, cell lysate ADPR, analyzed in the other study, differs from cytoplasmic ADPR, since it includes also nuclear ADPR which is largely catalyzed by PARP1/ARTD1 and whose contribution to extranuclear ADPR remains to be investigated.

In this study, we also investigated the subcellular source of cyADPR. The cytoplasm consists of several compartments/organelles (e.g., mitochondria, endoplasmic reticulum, golgi, cytosol) that could contribute to cyADPR, each of them characterized by the presence of different enzymes regulating ADPR [23, 54, 55]. We observed that in breast, colon, lung, and ovarian carcinomas the major source of cyADPR was the mitochondrion. However, residual ADPR signal was observed not to colocalize with this specific organelle. Thus, in order to precisely understand cyADPR distribution in the cytoplasm, a future colocalization study should be considered. Defining the exact localization of all cyADPR will allow to further investigate the molecular mechanism regulating cyADPR levels and the various patient prognosis. Additional mechanistic data might come from mass spectrometry analysis of the ADP-ribosylome in biopsies displaying different cyADPR staining intensity.

In this comprehensive study, we considered different cancer types: breast, ovarian, colon, lung, prostate and brain. Although for brain and colon cancer patient survival data were not available, in breast and high grade serous ovarian cancer cyADPR correlated with patient OS, whereas no statistically significant association was found for prostate and lung cancer. The reason for this discrepancy might be the limited number of cases available for the analysis in some tumor (sub)types (i.e., lung squamous and adenocarcinoma). Thus, for potential follow-up studies larger patient cohorts are needed to assess the prognostic value of cyADPR in other tumor types.

The anti-ADPr antibody used for the IHC stainings presented in this study detected ADPR, but did not allow to distinguish between MAR or PAR. For future studies, it would be of great interest to understand the prognostic impact of these two ADPR forms. Whether the determination of ADPR patterns and forms in aggressive tumors would also provide a rational for a therapeutic intervention remains to be investigated, especially since the currently available PARPi target mainly the nuclear ADPR and no other ART family members.

In summary, we provide evidence that the pattern of cyADPR implies an important role for cyADPR in the behavior of specific tumor types. Our data might be important for the understanding of different responses to PARPi therapy. In this context, it is tempting to speculate whether, in addition to “BRCAness”, the assessment of nuclear and/or cytoplasmic ADPR levels represents a predictive diagnostic tool for cancer therapy with PARPi.

## Supplementary information

Supplementary Figures

## Data Availability

All data is available in the main text or the supplementary materials. Raw data and materials are available upon request for non-commercial research purposes.
