# Supplementary material for: Cytoplasmic ADP-ribosylation levels correlate with markers of patient outcome in distinct human cancers
Source: Mod Pathol. 2021 Mar 19;34(8):1468–77. doi: 10.1038/s41379-021-00788-9 (PMC8295037; doi:10.1038/s41379-021-00788-9)
Supplement: Supplementary file 1 — Supplementary Figures [file 41379_2021_788_MOESM1_ESM.pdf]

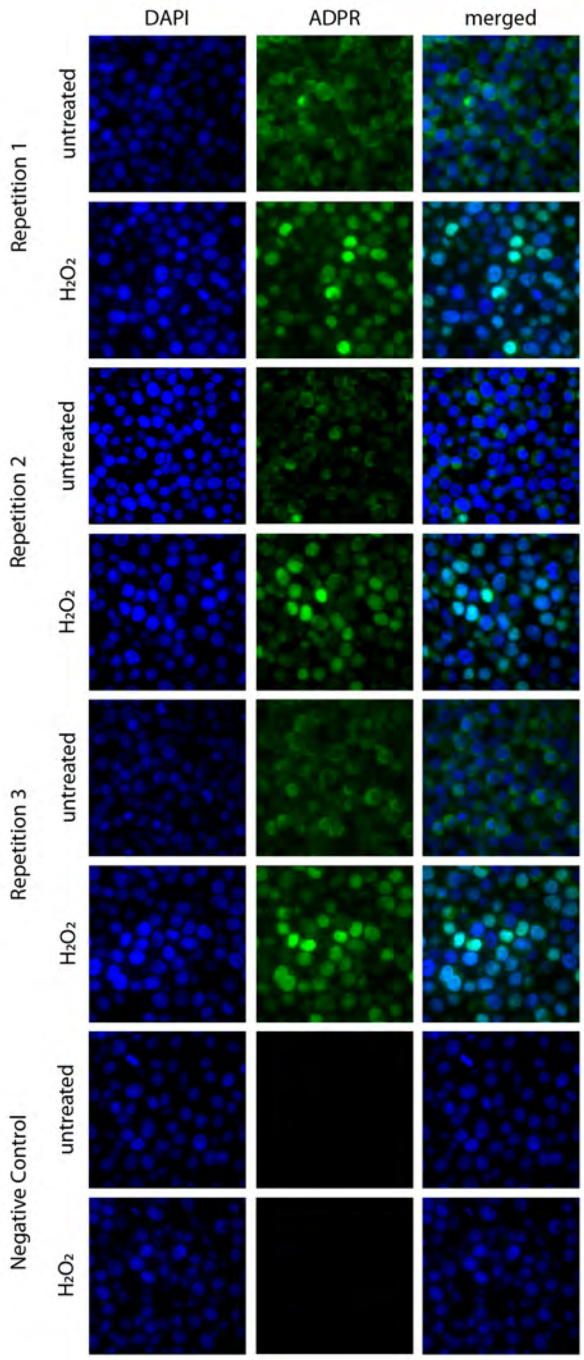

Supplementary Figure 1. Repeatability assay IF staining with anti-ADPr antibody on cell pellets. IF staining of HeLa cell pellets using the anti-ADPr antibody. HeLa cells untreated on the top and H_2O_2 treated on the bottom. Three repetitions performed in different days. Nuclear signal (DAPI, blue), ADPR signal (anti-ADPr antibody, green). Magnification 63x. Negative control stainings performed without the primary antibody.

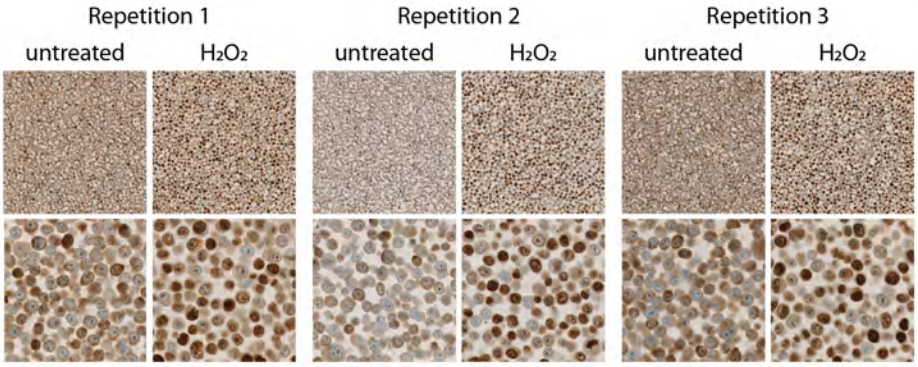

Supplementary Figure 2. Repeatability assay ICC staining with anti-ADPr antibody on cell pellets. ICC staining of HeLa cell pellets using the anti-ADPr antibody. HeLa cells untreated on the left and H_2O_2 treated on the right. Repetitions performed in three different days. Magnification 10x (top images) and 40x (bottom images).

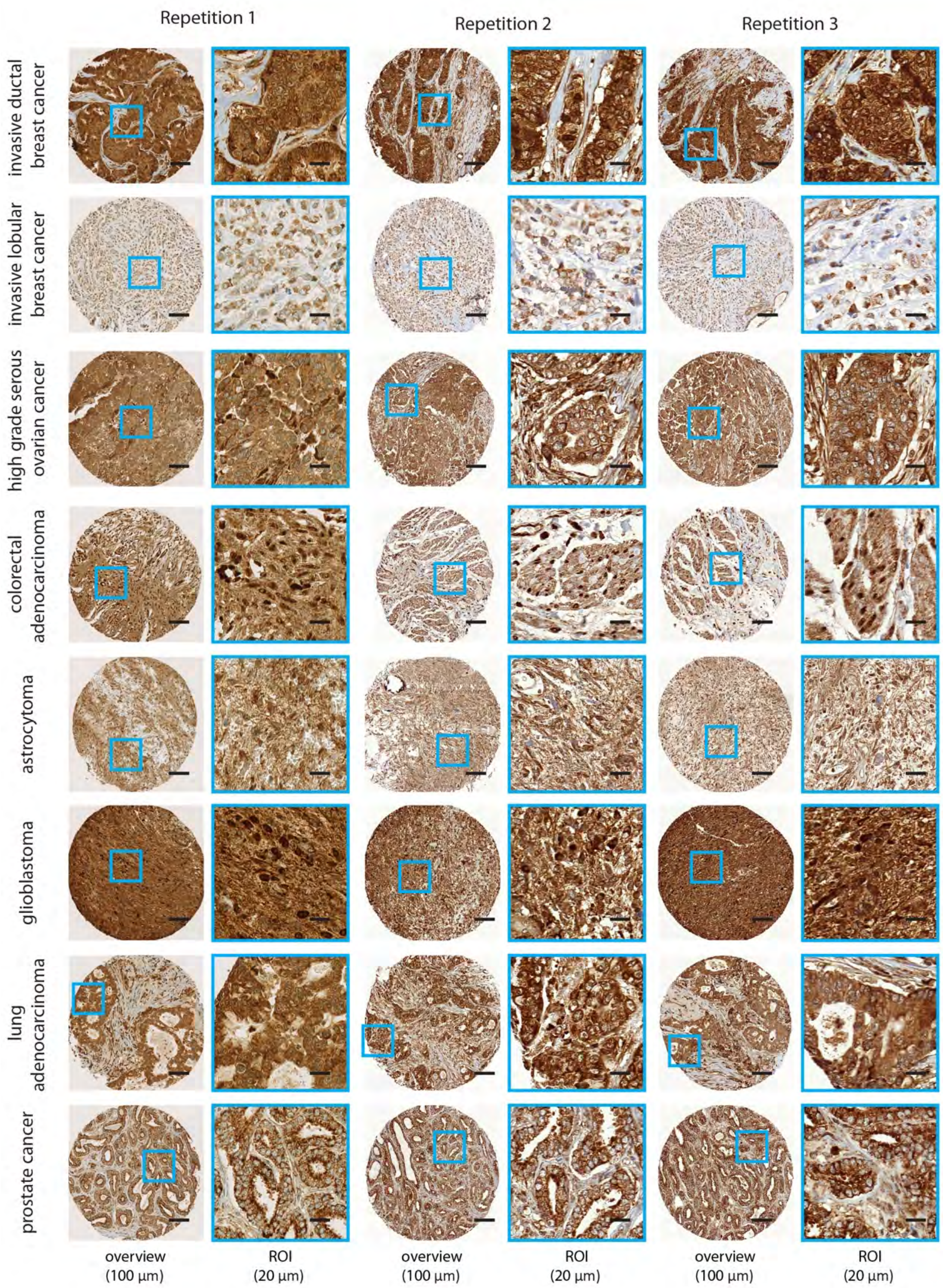

Supplementary Figure 3. Repeatability assay IHC staining with anti-ADPr antibody on tumor tissues. IHC staining of different tumor tissues included in a test TMA using the anti-ADPr antibody. Repetitions performed in three different days. Scale bar: overview 100 µm and ROI 20 µm.

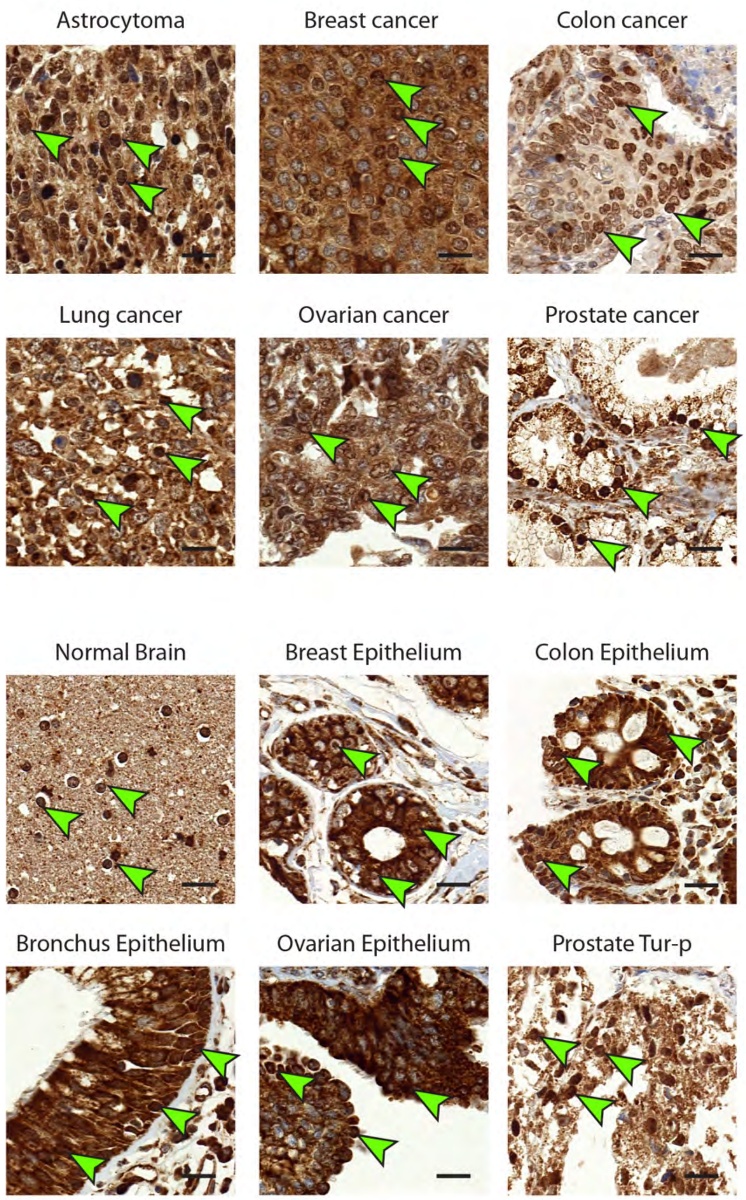

Supplementary Figure 4. Nuclear ADPR signal in cancer and healthy tissues. IHC staining of cancer, healthy and borderline biopsies using the anti-ADPr antibody. In the ROIs (scale bar 20 µm, 40x) nuclear ADPR is indicated by arrows.

Supplementary Figure 5. Analysis of the ADPR signal intensities in prostate cancer

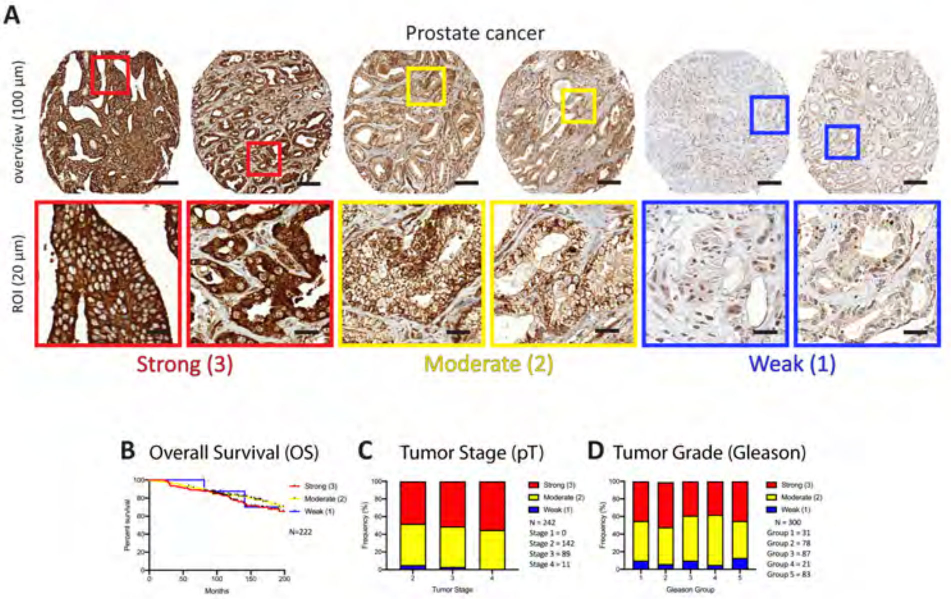

Supplementary Figure 5. Analysis of the ADPR signal intensities in prostate cancer. **A)** IHC staining of a prostate cancer TMA using the anti-ADPr antibody. Staining intensity scores are represented as strong (red, 3), moderate (yellow, 2) and weak/negative (blue, 1). On the top, TMA cores (scale bar 100 µm, 10x), on the bottom, ROIs (scale bar 20 µm, 40x). **B**) Kaplan-Meier survival plot (Mantel-Cox test, N=222) with patients stratified based on the cyADPR signal intensity scores. **C**) Contingency analysis to assess the association between cyADPR scores and the tumor stage (pT 1-4) (chi-squared test, N=242). **D**) Contingency analysis to assess the association between cyADPR scores and the tumor grade (1-3) (chi-squared test, N=300). Gleason grades are represented in five groups, as follow: group 1 (Gleason score <6), group 2 (Gleason score 7 (3+4)), group 3 (Gleason score 7 (4+3)), group 4 (Gleason score 8) and group 5 (Gleason score >9).

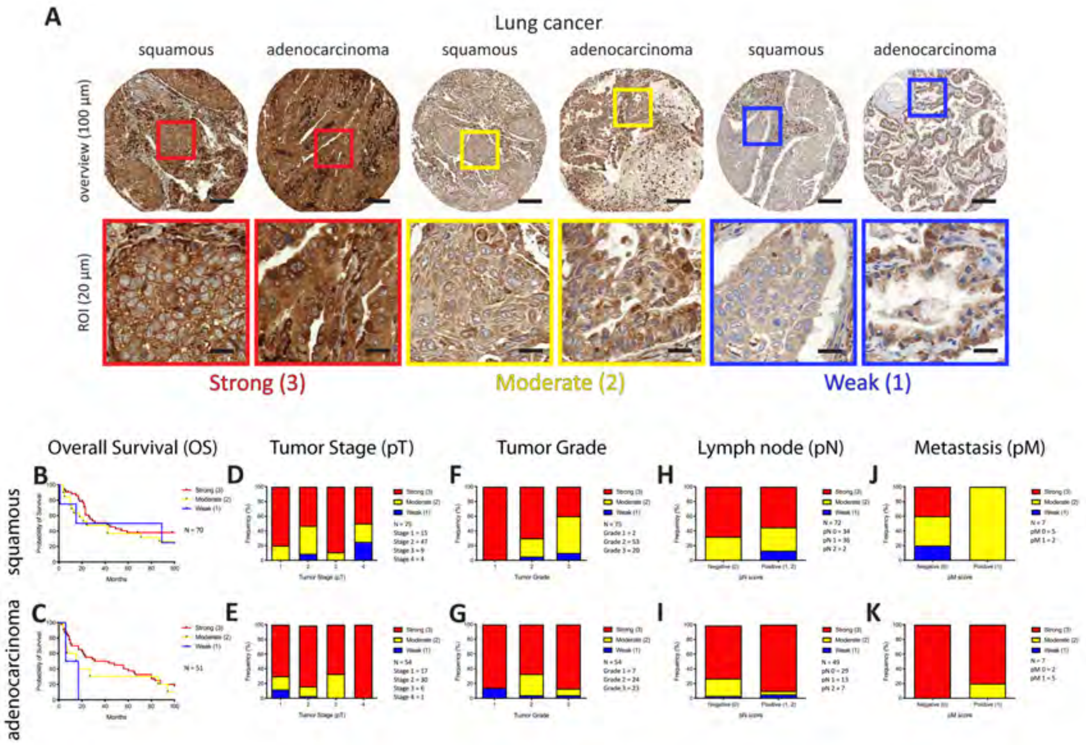

Supplementary Figure 6. Analysis of the ADPR signal intensities in lung cancer. **A)** IHC staining of a lung cancer TMA using the anti-ADPr antibody. Staining intensity scores: strong (red, 3), moderate (yellow, 2) and weak/negative (blue, 1). On the top, TMA cores (scale bar 100 µm, 10x), on the bottom, ROIs (scale bar 20 µm, 40x). Kaplan-Meier survival plot of **B**) squamous (Mantel-Cox test, N=70) and **C)** adenocarcinoma lung cancer (Mantel-Cox test, N=51) with patients stratified based on the cyADPR signal intensity scores. Contingency analysis to assess the association between cyADPR scores and the tumor stage (pT 1-4) in **D**) squamous (chi-squared test, N=75) and in **E**) adenocarcinoma lung cancer (chi-squared test, N=54). Contingency analysis to assess the association between cyADPR scores strong (red, 3), moderate (yellow, 2), weak/negative (blue, 1) and the tumor grade (1-3) in **F**) squamous (chi-squared test, N=75) and in **G**) adenocarcinoma lung cancer (chi-squared test, N=54). Contingency analysis to assess the association between cyADPR scores and the presence of regional lymph node metastasis (pN 0-2) in **H**) squamous (chi-squared test, N=72) and in **I**) adenocarcinoma lung cancer (chi-squared test, N=49). Contingency analysis to assess the association between cyADPR scores and the presence of distant metastasis (pM 0-1) in **J**) squamous (chi-squared test, N=7) and in **K**) adenocarcinoma lung cancer (chi-squared test, N=7).

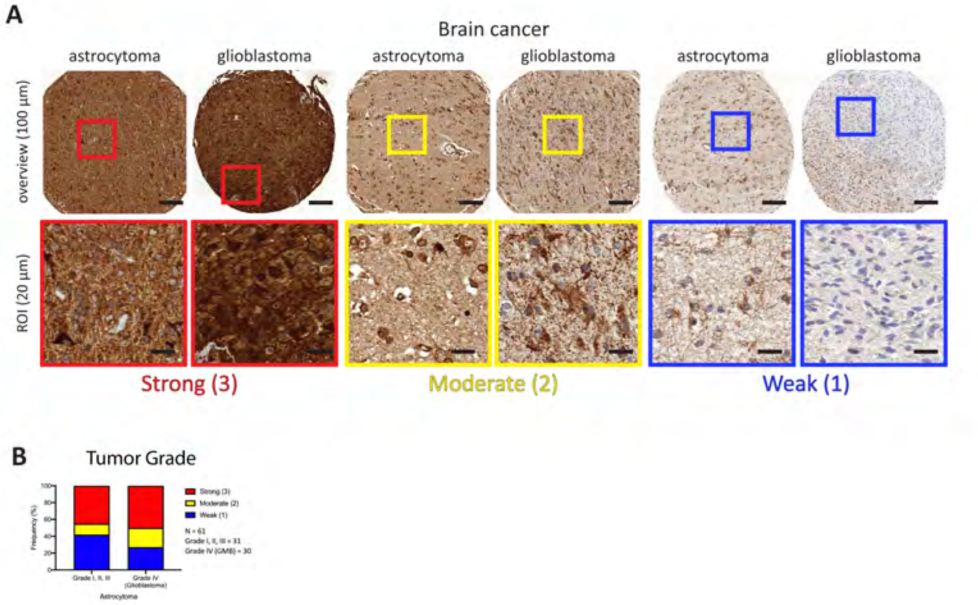

Supplementary Figure 7. Analysis of the ADPR signal intensities in brain cancer. **A)** IHC staining of a brain cancer TMA using the anti-ADPr antibody. Staining intensity scores: strong (red, 3), moderate (yellow, 2) and weak/negative (blue, 1). On the top, TMA cores (scale bar 100 µm, 10x), on the bottom, ROIs (scale bar 20 µm, 40x). **B**) Contingency analysis to assess the association between cyADPR scores and the astrocytoma grade I-III vs glioblastoma grade IV (chi-squared test, N=61).

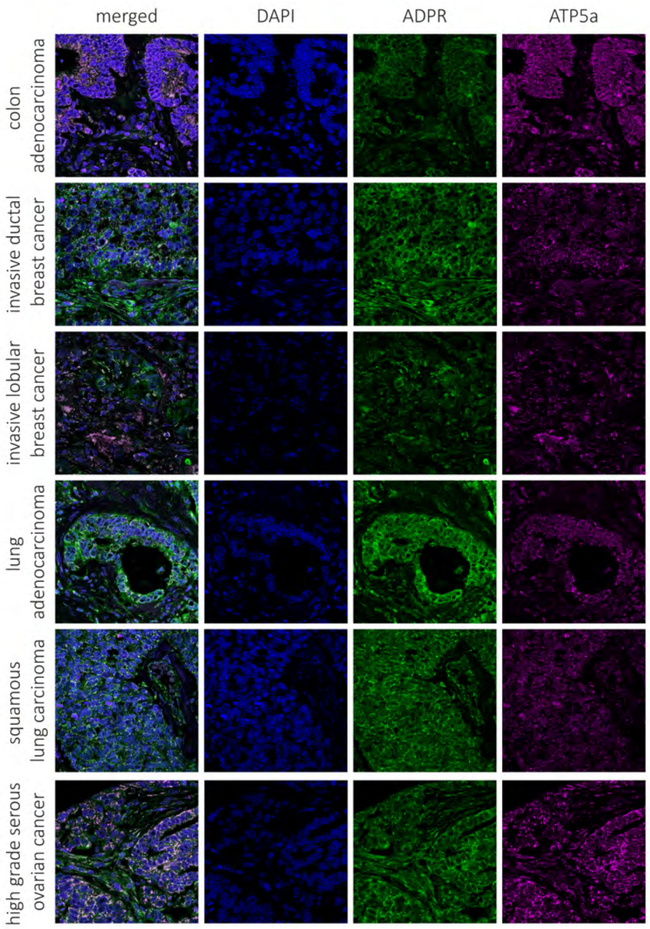

Supplementary Figure 8. Co-localization of ADPR and ATP5a in tumor tissues. Confocal microscope images (63x magnification) of FFPE patient tissue biopsies stained with DAPI (blue), ADPR (green) and ATP5a (pink).
